# An Assessment of Surgical Outcomes in Malignant Peripheral Nerve Sheath Tumors: A Systematic Review and Meta-Analysis of Surgical Interventions

**DOI:** 10.3390/cancers17121997

**Published:** 2025-06-15

**Authors:** Abdel-Hameed Al-Mistarehi, Khaled J. Zaitoun, Jawad Khalifeh, Max A. Saint-Germain, Melanie Alfonzo Horowitz, Abdul Karim Ghaith, Chase H. Foster, Shoshana Braverman, Avi N. Albert, Usama AlDallal, Allan Belzberg, Sang Lee, Nicholas Theodore, Ilya Laufer, Daniel Lubelski

**Affiliations:** 1 Department of Neurosurgery, Johns Hopkins School of Medicine, Baltimore, MD 21287, USA; aalmist1@jh.edu (A.-H.A.-M.); jkhalif1@jhmi.edu (J.K.); msaintg1@jhmi.edu (M.A.S.-G.); malfonz1@jhu.edu (M.A.H.); aghaith1@jh.edu (A.K.G.); cfoste43@jh.edu (C.H.F.); slee439@jh.edu (S.L.); theodore@jhmi.edu (N.T.); 2 Faculty of Medicine, Jordan University of Science and Technology, Irbid 22110, Jordan; kjzaitoun20@med.just.edu.jo; 3 Department of Neurosurgery, School of Medicine, George Washington University, Washington, DC 20037, USA; 4 Stern College for Women, Yeshiva University, New York, NY 10033, USA; sbraver1@mail.yu.edu; 5 School of Medicine, Meharry Medical College, Nashville, TN 37208, USA; aalbert24@mmc.edu; 6 School of Medicine, Medical University of Bahrain, Adliya P.O. Box 15503, Bahrain; ualdallal@gmail.com; 7 Department of Neurosurgery, New York University Langone Health, New York, NY 10016, USA; ilya.laufer@nyulangone.org

**Keywords:** MPNST, malignant peripheral nerve sheath tumors, survival, progression-free survival, PFS, recurrence, mortality

## Abstract

Background/Objectives: Malignant peripheral nerve sheath tumors (MPNSTs) are aggressive malignancies with a challenging prognosis, especially for patients with Neurofibromatosis type 1 (NF1). Their low incidence necessitates comprehensive studies to investigate the survival outcome. Methods: We conducted a systematic review and meta-analysis, including data from 16 studies and 4265 patients, to explore surgical outcomes and survival rates, focusing on time-related outcomes, including overall survival (OS), progression-free survival (PFS), and recurrence rate. Results: The analysis revealed that the OS rate was 86% [95% CI: 75–97%] at 1 year, decreasing to 60% [95% CI: 45–75%] at 3 years, and further declining to 47% [95% CI: 35–58%] by 5 years. For PFS, the 1-year rate was 61% [95% CI: 25–98%], which remained similar at 62% [95% CI: 35–89%] for 3 and 5 years. In NF1-associated MPNSTs, the 1-year OS was relatively high at 93% [95% CI: 83–100%], but it dropped to 68% [95% CI: 53–84%] at 3 years and further to 50% [95% CI: 31–68%] at 5 years. Additionally, the hazard ratio indicated a 38% lower survival rate in NF1 patients than those with sporadic MPNSTs when data were presented in the same study. Recurrence rates were high, with 56% of patients experiencing a relapse, primarily as local recurrences (70.6%). Mortality was significant, with over 50% of patients dying within an average follow-up period of 33.45 months. Conclusions: MPNSTs, particularly in NF1 patients, are associated with poor prognosis and high recurrence rates. These results underline the necessity of targeted therapeutic strategies and improved programs for screening, mainly through a multidisciplinary approach to optimize management.

## Simple Summary

Malignant peripheral nerve sheath tumors (MPNSTs) are rare soft tissue sarcomas with peripheral nerve sheath differentiation, which tend to be locally aggressive, with systemic metastasis and poor survival outcomes. Although we understand MPNST pathophysiology, less is known about the time-to-event metrics, such as mortality, progression-free survival, and overall survival. In this study, we sought to characterize these time-to-event metrics and survival outcomes by conducting a thorough systematic review of published studies of surgically managed MPNST. Understanding these metrics will emphasize the need for targeted therapeutic strategies and improved screening programs for patients with MPNST and encourage multidisciplinary collaboration to optimize management of these tumors.

## 1. Introduction

Malignant peripheral nerve sheath tumors (MPNSTs), as malignant soft tissue sarcomas with peripheral nerve sheath differentiation, are rare. They occur sporadically in 1 in 100,000 among the general population but at higher rates of 1.6 in 1000 among individuals with an underlying genetic predisposition, namely neurofibromatosis type 1 (NF1) [1,2]. Patients often present with pain, sensory symptoms, or neurologic compromise due to local neural invasion or mass effect. MPNSTs can be locally aggressive, with a tendency for systemic metastasis in up to 70% of patients and poor survival outcomes [3,4]. Wide surgical resection, with a negative margin in all directions, when possible, is the mainstay of treatment for localized disease, as these tumors are not particularly sensitive to chemo- or radiotherapy [5,6].

Studies have improved our understanding of MPNST pathophysiology, diagnosis, and multidisciplinary management strategies; there remains a need for better analysis of time-to-event metrics, such as mortality, progression-free survival (PFS), disease-specific survival (DSS), and overall survival (OS) [7,8]. In a 2020 study of 115 patients with advanced or metastatic MPNST in any location who received various chemotherapeutic options, Sobczuk et al. found a median OS of 15 months with a one-year OS rate of 63% [9]. In a 2011 study of 16 patients with spinal MPNST, Zhu et al. found a one-year OS of 48% [10]. Differences in reported survival outcomes can be attributed to patient-specific factors, such as sex, age, and NF1 status, and tumor-related factors, such as size, location, grade, genetics, systemic treatment, and extent of surgical resection.

We propose a thorough literature review of time-to-event metrics across all published studies of surgically managed MPNST to mitigate institutional and patient-level differences and small sample sizes [11]. In this systematic review, we aim to investigate the effects of demographics, clinical history, and genetic predisposition syndromes (e.g., NF1) on survival outcomes and time-to-event metrics among surgically managed patients with MPNST.

## 2. Materials and Methods

### 2.1. Data Sources and Search Strategy

This study is a systematic review of the literature pertaining to the surgical management of MPNSTs. The published literature was reviewed following the PRISMA guidelines. Our search strategy used a combination of the following keywords and Boolean operators: “((malignant peripheral nerve sheath tumor) OR (MPNST)) AND ((surgery) OR (surgical))”. The search strategies were implemented in PubMed MEDLINE in February 2024.

### 2.2. Eligibility Criteria

We reviewed English-language articles using the following PICOS criteria: (P) Population: Adult patients (≥16 years old) of both sexes with primary or metastatic MPNST. (I) Intervention: Surgical intervention. (C) Comparator: None. (O) Outcomes: mortality, PFS, OS, DSS, recurrence rate, and signs and symptoms. Our inclusion criteria further included only original research reporting primary data on human participants. We included case series, cohort studies, and case–control studies, while we excluded literature reviews, technical notes, abstracts, autopsy reports, case reports, systematic reviews, and meta-analyses.

### 2.3. Study Selection

Citation titles and abstracts were screened in a blinded manner by two reviewers for prespecified selection criteria. This was followed by a full-text review of the remaining articles for eligibility. Any disagreement was resolved through discussion or by involving a third reviewer. The included studies were reported in a PRISMA flow diagram.

### 2.4. Data Extraction

Three independent investigators extracted data from the included studies. We collected information on article title, author, journal, publication year, country of origin, study design, total participants, main inclusion criteria, primary outcome, and follow-up duration. We extracted patient demographics (age, sex) and MPNST characteristics (etiology, size, location, single or multiple, signs and symptoms, diagnostic tests). We extracted information on treatment approaches (nonoperative, operative) and outcomes (mortality rates, recurrence rates, complications related to treatment, PFS, OS, DSS, and extent of resection). We extracted Kaplan–Meier survival curves and survival data for each time point (1 year, 3 years, and 5 years) [12].

### 2.5. Statistical Analysis

All analyses were performed using RStudio version 2023.06.1 + 524 [13] and R version 4.2.1. Descriptive statistics summarized patient-level data on demographics, clinical features, and operative characteristics. Time-to-event outcomes are presented as the true (pooled) estimate proportions for the general population by pooling studies that reported total patient outcomes (without NF1 stratification) and for the NF1 subgroup analysis by including the studies that stratified their results by NF1 status. Also, the outcomes were compared between the syndromic vs. sporadic cases using studies that reported the hazard ratio (HR) to compare the two groups. The respective 95% CIs were calculated for each estimate and presented in forest plots. When HR was not reported in the study, we calculated the HR using methods previously described by Tierney et al. [14].

Heterogeneity was assessed using the I^2^ and chi-square (χ^2^) statistics. When I^2^ < 50%, it was considered low heterogeneity, whereas I^2^ ≥ 50% indicated substantial heterogeneity. The χ^2^ test was considered statistically significant with a *p*-value less than 0.05. We used a fixed-effect model if no significant heterogeneity was present; otherwise, we used a random-effect model.

The possibility of publication and selection bias was assessed using visual inspection of the funnel plot and Egger’s test. Notably, these methods were initially developed for use in comparative studies [15].

### 2.6. Ethical Oversight

IRB approval was not required or sought for this systematic review, as it involved secondary analysis of published de-identified data. The study protocol has not been registered.

## 3. Results

### 3.1. Search Results and Study Selection

**Figure 1:**
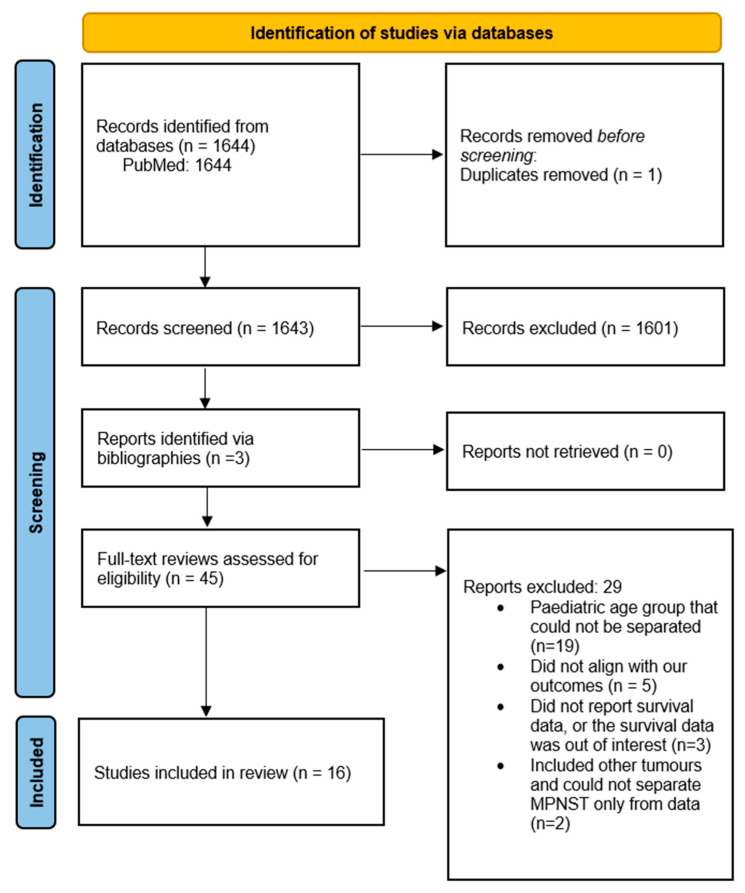
PRISMA flow chart of the included studies.

Our search of the literature yielded 1644 results. One duplicate was excluded. We screened the titles and abstracts of 1643 articles, from which we were able to exclude 1601 reports. The full texts of the 45 remaining articles were reviewed. After a full-text review, 16 total studies were included (Figure 1). Supplementary Table S1 details which studies were eligible for inclusion in the pooled analyses of survival outcomes and NF1 subgroup outcomes. Reasons for exclusion from each endpoint are summarized.

### 3.2. Demographic and Clinical Characteristics of Patients in the Reviewed Studies

A total of 4265 patients were included in the analysis. The average age (SD) was 46.91 (1.99) years. Greater than half of the cohort (53.7%) were men. Table 1 summarizes the demographic and clinical characteristics of the study cohort. Tumor characteristics showed an average size of 7.65 cm (SD ± 1.07). Of the 3434 patients with reported tumor grades, 69.8% presented with high grade MPNSTs, while the remaining 30.2% had low grade MPNSTs. Tumor locations were predominantly in the trunk/extremities (77.3%), followed by the head/neck (13.0%). Among 54 patients with documented pre-operative symptoms, 79.7% presented with a palpable/growing mass, and 61.3% reported pain. NF1 was identified in 32.0% of 1375 patients, while metastasis was observed in 17.0% of 4109 patients. Surgical resection was performed in 68.5% of cases (*n* = 2354), with gross total resection achieved in 72.6% of them (*n* = 1708).

Of the ten studies and 3342 patients with mortality outcomes data, the mortality rate was 50.4% (*n* = 1685) over a mean follow-up duration of 33.53 months (SD ± 16.34). Eleven studies reported recurrence in 56.0% of 445 patients, with local recurrence in 70.6% and distal recurrence in 29.4%. Detailed study characteristics can be found in Supplementary Table S2.

### 3.3. Proportional Survival Results

Table 2 summarizes the PFS, OS, and DSS results of the proportional meta-analysis. A random-effect model was used across all analyses due to high heterogeneity (I^2^ > 50% or *p* < 0.05). At 1 year, the pooled proportion of PFS was 0.61 (95% CI: 0.25–0.98) across four studies with 166 patients, showing high heterogeneity (I^2^ = 98%, *p* < 0.01) (Figure S1). At 3 and 5 years, the pooled proportions of PFS were the same with 0.62 (95% CI: 0.35–0.89) from two studies with 41 patients, with significant heterogeneity (I^2^ = 72%, *p* = 0.06) (Figures S2 and S3).

**Figure 2:**
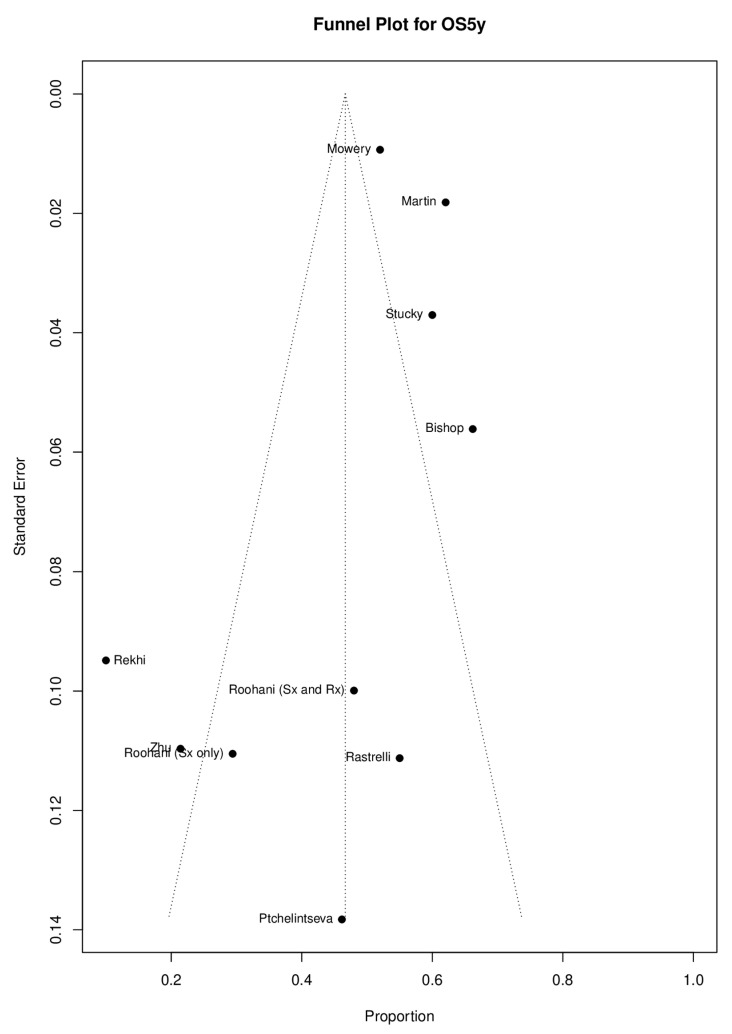
Funnel plot for overall survival and disease-specific survival at 5 years.

At 1 year, the pooled proportion of OS was 0.86 (95% CI: 0.75–0.97) from eight studies, including 3072 patients (I^2^ = 89%, *p* < 0.01) (Figure S4). At 3 years, it was 0.60 (95% CI: 0.45–0.75) from six studies with 2947 patients (I^2^ = 80%, *p* < 0.01) (Figure S5). At 5 years, the pooled proportion of OS was 0.47 (95% CI: 0.35–0.58) from ten studies with 3917 patients, showing significant heterogeneity (I^2^ = 87%, *p* < 0.01) and significant subgroup difference between OS (0.47, 95% CI: 0.35–0.58) and DSS (0.46, 95% CI: 0.35–0.58) (*p* = 0.04) (Figure S6). Although the Egger’s test result (95% CI: −3.13–1.49, *p* = 0.507) does not indicate the presence of funnel plot asymmetry, visual inspection of the funnel plot suggests otherwise (Figure 2).

### 3.4. NF-1 Subgroup

In the NF-1 cohort, the pooled proportion of OS and DSS at 1 year was 0.93 (95% CI: 0.83–1.00) from three studies with 120 patients (I^2^ = 77%, *p* = 0.01). However, the proportion for 1 year OS was 0.96 (95% CI: 0.87–1.00) from two studies, while the 1-year proportion of DSS was 0.81 (95% CI: 0.58–0.95), with no significant subgroup difference (*p* = 0.12) (Figure S7). At 3 years, the pooled proportion of DSS was 0.68 (95% CI: 0.53–0.84) from two studies with 99 patients (I^2^ = 65%, *p* = 0.09) (Figure S8). At 5 years, the pooled proportion of both OS and DSS was 0.50 (95% CI: 0.31–0.68) from five studies with 181 patients, showing significant heterogeneity (I^2^ = 86%, *p* < 0.01). The proportions for OS and DSS at 5 years were 0.63 (95% CI: 0.53–0.72) and 0.57 (95% CI: 0.34–0.77), respectively, with no significant difference (*p* = 0.13) (Figure S9).

### 3.5. NF-1 vs. Sporadic Cases

**Figure 3:**
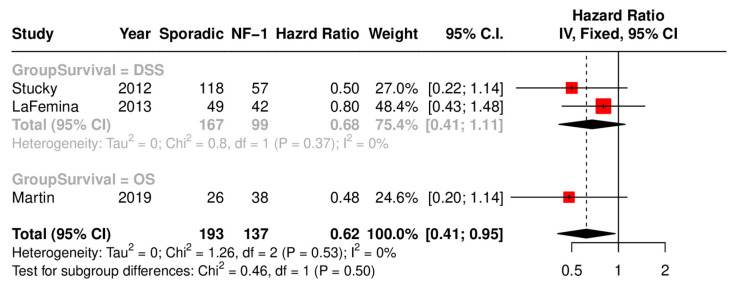
Forest plot comparing the overall survival between syndromic and sporadic cases.

The comparison of sporadic and NF1 patients using a fixed-effect model (I^2^ = 0%, *p* = 0.53) showed worse OS outcomes associated with NF-1-associated MPNSTs. Figure 3 demonstrates the survival outcomes of the studies, including comparison of NF1 with sporadic cases. The overall HR is 0.62 (95% CI: 0.41, 0.95), showing that the NF1 patients had lower survival rates than sporadic cases.

## 4. Discussion

Malignant peripheral nerve sheath tumors (MPNSTs) are locally aggressive soft tissue sarcomas and can be associated with poor outcomes. These tumors are rare in the general population; however, they are enriched among patients with NF1 who show malignant transformation of previously benign neurofibromas. The rarity of this disease lends to a paucity of data on outcomes related to OS or PFS in the context of surgical resection. While Cai et al. (2020) published a review paper on prognostic factors for MPNSTs, this study did not focus on MPNST surgical outcomes or related survival metrics, such as overall survival (OS), progression-free survival (PFS), and recurrence rates [16]. Additionally, this review only includes data up to February 2020 and broadly analyzes prognostic factors, without providing specific details regarding surgical outcomes [16]. In this study, we address this gap by systematically summarizing and synthesizing existing articles investigating surgical outcomes in patients with MPNST. In our query, we identified 16 studies with a combined 4265 patients. In a subsequent analysis, we divide the patient population into those who developed NF1-associated MPNSTs versus sporadic cases to analyze differences in the two cohorts. Unlike Cai et al., who only broadly mentioned NF1 as a prognostic factor, our study conducts a subgroup analysis between NF1-associated and sporadic MPNSTs, providing a direct comparison and insights into survival differences between these groups [16].

### 4.1. Survival and Clinical Features

Martin et al. reported a median survival of six years among the adult MPNST patients who underwent resection [17], whereas our results suggest a mean survival of 33.45 ± 16.37 months, less than three years. At 5 years, the OS rate is low, ranging between 20 and 50% [10,18,19]. Our meta-analysis results are concordant with the literature, as our results demonstrated a 3- and 5-year survival rate of 60% and 47%, respectively.

Surgical resection with wide margins is currently the gold standard in curative MPNST treatment. Neoadjuvant therapy falls short as these tumors are particularly chemotherapy-resistant, as compared to other soft tissue sarcomas, and the efficacy of high-dose radiation therapy is questionable [20,21,22,23,24]. Factors shown to be associated with poor prognosis are large tumor size, positive resection margins, and central/axial tumor location [25]. A negative surgical margin and a tumor diameter of less than 5 cm are associated with an improved prognosis [26,27]. In our review, the average tumor size was 7.65 cm, and they were primarily located in the truncal region. These tumors, according to Stucky et al. [26] and Anghileri et al. [22], are associated with poor outcomes, owing to the relationship of these large tumors with nearby vital neurovascular structures that would limit the ability for a negative surgical margin without causing undue morbidity and mortality [28]. Similarly to our data, most MPNSTs present high-grade sarcomas, which have a strong correlation with reduced survival outcomes [7,29].

### 4.2. Tumor Relapse and Recurrence

MPNSTs have the highest recurrence rate of all soft tissue sarcomas [30]. In our study, at 1, 3, and 5 years, the tumor recurrence rates were 61%, 62%, and 62%, respectively. This is consistent with the available literature, which reveals that the local recurrence rate for a patient who received an MPNST surgical resection is between 40 and 70% [10,31,32]. Despite negative surgical margins and neoadjuvant therapy consisting of chemotherapy or radiation, there is still a high likelihood of MPNST recurrence. It is more common for MPNSTs to recur locally rather than metastasize [27]. Local recurrences present added morbidity, as patients have already undergone multimodal operative and nonoperative therapy, distorting the local anatomy and limiting safe repeat surgical resection [33].

Anghileri et al. reported a 2.4-increased risk of developing a local recurrence in patients whose pathology reports positive margins [22]. Additionally, MPNSTs located near vital neurovascular structures, such as those found in the cranial region, present with increased difficulty obtaining a complete resection and may lead to a higher likelihood of local recurrence [34]. Overall, Cao et al. found that the factors associated with MPNST local recurrence are tumor size, tumor site, and margins, like those of survival [35]. Metastatic recurrence occurs less frequently, though it is associated with worse outcomes and frequently occurs in the lungs [21].

### 4.3. NF-1 vs. Sporadic Cases

Most MPNSTs occur in NF-1 patients [1]. Non-NF1 patients may develop MPNSTs sporadically or due to malignant transformation of benign nerve sheath tumors. Although the proportional meta-analysis suggests that NF-1-associated MPNSTs seem more favorable regarding survival than all cases, the head-to-head comparison of NF-1 to sporadic patients showed that NF-1 status confers lower survival rates than sporadic MPNST. The literature regarding the effect of NF-1 on MPNST prognosis is inconclusive. A prior meta-analysis by Puhaindran et al. [36] demonstrated that NF-1 is associated with an increased mortality risk in MPNST patients. Other studies have shown similar results. However, this finding is challenged by different studies that found no such association [22,37].

In their series of 134 patients, Gunderson et al. explain a worse prognosis in NF-1 patients histologically, as the NF-1-associated tumors have larger tumors with increased mitoses per high power field [21]. Tabone-Eglinge et al. describe that NF-1 MPNSTs are likelier to be Epidermal Growth Factor Receptor (EGFR)-mRNA-positive than other MPNSTs [38]. In their series, a high EGFR expression was reported in highly cellular areas of the tumors. Gronchi et al. argue that the difference in survival between NF-1 and non-NF-1 MPNST patients is due to NF-1 patients presenting later with larger tumors due to a failure of recognition of malignancy within large plexiform neurofibromas, and there is no biological basis for the difference in survival. Although the evidence for genetic hallmarks of NF-1-associated MPNST is limited, this theory should not be ruled out, as future therapeutics may potentially rely on distinct molecular targets to improve survival and relapse rates.

### 4.4. Heterogeneity in Survival Outcomes

Across the survival endpoints we examined, including overall and progression-free survival, our meta-analyses showed marked statistical heterogeneity (I^2^ > 70–90%). This appears to reflect both clinical and methodological variation among studies. Clinically, cohorts differed in patient demographics (e.g., NF1-related vs. sporadic cases), tumor size, grade, location, and treatment strategies (margin status, radiotherapy, and chemotherapy). Methodologically, discrepancies in study design, reporting quality, follow-up length, and outcome definitions likely compounded the variability. We employed a random-effects model to account for these differences, yet the remaining heterogeneity highlights the need for more standardized reporting and, where feasible, individual-patient-data meta-analyses in future work.

Notably, the estimates for PFS at 3 and 5 years are derived from only two studies with a total of 41 patients, which further compounds heterogeneity and limits the statistical power and generalizability of these findings. While the pooled estimates provide a preliminary indication of outcomes at these time points, they should be interpreted cautiously due to the small sample size and high heterogeneity (I^2^ = 72%).

### 4.5. Study Limitations

Our systematic review and meta-analysis have several limitations. First, our data reveal a significant degree of heterogeneity in published studies. This can be explained by the rarity of MPNSTs and scattered treatment paradigms. Nonetheless, systematically pooling and analyzing these data is crucial to understanding these tumors and informing proper management strategies. This effect was minimized through the random-effect model. Second, although all patients in our analysis underwent surgical resection, they differ in the extent of resection and perioperative systemic therapy. Third, several subgroup analyses, such as PFS at 3 and 5 years, were based on a limited number of studies (*n* = 2) and small sample sizes (<50 patients). Although we pooled these data to provide the most comprehensive synthesis possible in this rare tumor population, the resulting estimates are inherently less reliable and should be interpreted cautiously. Future studies with larger sample sizes are needed to validate these outcomes. Forth, the tumor-site distribution is based on incomplete reporting; the true proportions across all patients may differ. Lastly, all information was collected retrospectively, subject to reviewer bias, loss of follow-up, and confounders. Although it is the best option for objective results, a randomized control trial is impractical for MPNSTs due to their low prevalence in the general population.

## 5. Conclusions

This systematic review and meta-analysis highlight the poor prognosis associated with MPNSTs, particularly in syndromic patients. Despite surgical interventions, high recurrence and mortality rates persist. Notably, NF1-associated cases show significantly worse outcomes compared to sporadic cases. These findings underscore the need for targeted therapeutic strategies, improved screening programs for MPNSTs, and a multidisciplinary approach to optimize the management and outcomes of these rare tumors.

## Figures and Tables

**Table 1:** Summary statistics of demographics and clinical features of the studied patients (*n* = 4253).

	Number of Studies	Sample Size	Frequency, (%) Within the Sample Size
Age (years); M ± SD	15	3539	46.91 ± 1.99
Sex	15	4234	
Male			2284 (53.7)
Female			1950 (46.3)
Tumor Characteristics			
Size (cm^3^); M ± SD	8	3258	7.65 ± 1.07
Tumor Location			
Trunk/Extremity	14	4191	3239 (77.3)
Trunk	13	1333	553 (41.5)
Extremity	12	1310	567 (43.3)
Head/Neck	12	4177	543 (13)
Other	4	3099	414 (13.4)
Grade	11	3434	
Low			1038 (30.2)
High			2396 (69.8)
Pre-op Symptoms			
Mass	3	54	43 (79.7)
Pain	2	44	27 (61.4)
Neurofibromatosis-1 (NF-1)	14	1375	438 (32.0)
Metastasis on presentation	13	4109	699 (17.0)
Treatment Protocol	14	3434	
Surgery			2354 (68.5)
Gross Total Resection	12	2354	1708 (72.6)
Chemotherapy			1739 (50.6)
Radiation			1763 (51.3)
Outcomes			
Tumor Recurrence	11	445	249 (56.0)
Local			176 (70.6)
Distant			73 (29.4)
Mean Follow-up; M ± SD	10	3423	33.53 ± 16.34
Mortality Rate	10	3342	1685 (50.4)

Percentages are calculated using only those studies that provided data for the given variable; totals therefore vary across rows.

**Table 2:** Summary of proportional meta-analyses of survival outcomes among the included studies.

	Studied Outcome	End-Point, Years	No. of Patients	# of Studies	Pooled Proportion	95% CI	I^2^	*p*-Value	*p*-Value for Subgroup Difference
**Progression-Free Survival**	PFS	1	166	4	0.61	[0.25, 0.98]	98%	<0.01	-
	PFS	3	41	2	0.62	[0.35, 0.89]	72%	0.06	-
	PFS	5	41	2	0.62	[0.35, 0.89]	72%	0.06	-
**Patient Survival**	OS	1	3072	8	0.86	[0.75, 0.97]	89%	<0.01	-
	OS	3	2947	6	0.60	[0.45, 0.75]	80%	<0.01	-
	OS and DSS	5	3917	10	0.47	[0.35, 0.58]	87%	<0.01	**0.04**
**Neurofibromatosis-1 (NF-1) Cohort**									
**Patient Survival**	OS and DSS	1	120	3	0.93	[0.83, 1.00]	77%	0.01	0.12
	DSS	3	99	2	0.68	[0.53; 0.84]	65%	0.09	-
	OS and DSS	5	181	5	0.50	[0.31, 0.68]	86%	<0.01	0.13

Abbreviations: PFS, progression-free survival; OS, overall survival; DSS, disease-specific survival.

## Data Availability

No new data were created or analyzed in this study. Data sharing is not applicable to this article.

## Supplementary Materials

The following supporting information can be downloaded at https://www.mdpi.com/article/10.3390/cancers17121997/s1, Table S1: Summary of Study Eligibility for Overall Survival Endpoints and NF1 Stratification; Table S2: Demographics, tumor characteristics, presentation, treatment, and outcomes in the included studies; Figure S1: Forest plot for the pooled proportion of patient’s survival using progression-free survival (PFS) at 1-year endpoint; Figure S2: Forest plot for the pooled proportion of patient survival using progression-free survival (PFS) at 3-year endpoint; Figure S3: Forest Plot for the pooled proportion of patient’s survival using progression-free survival (PFS) at 5-year endpoint; Figure S4: Forest plot for the pooled proportion of patient’s survival using overall survival (OS) at 1-year endpoint; Figure S5: Forest plot for the pooled proportion of patient’s survival using overall survival (OS) at 3-year endpoint; Figure S6: Forest plot for the pooled proportion of patient’s survival using overall survival (OS) and disease-free survival (DSS) at 5-year endpoint; Figure S7: Forest plot for the pooled proportion of NF-1 patients survival using overall survival (OS) and disease-free survival (DSS) at 1-year endpoint; Figure S8: Forest plot for the pooled proportion of NF1 patient’s survival using disease-specific survival (DSS) at 3-year endpoint; Figure S9: Forest plot for the pooled proportion of NF1 patient’s survival using overall survival (OS) and disease-specific survival (DSS) at 5-year endpoint.

## Abbreviations

The following abbreviations are used in this manuscript:

MPNST	Malignant Peripheral Nerve Sheath Tumors
NF1	Neurofibromatosis Type 1
PFS	Progression-free Survival
OS	Overall Survival
DSS	Disease-specific Survival
HR	Hazard Ratio
CI	Confidence Interval
EGFR	Epidermal Growth Factor Receptor
