# Supplementary material for: An Assessment of Surgical Outcomes in Malignant Peripheral Nerve Sheath Tumors: A Systematic Review and Meta-Analysis of Surgical Interventions
Source: Cancers (Basel). 2025 Jun 15;17(12):1997. doi: 10.3390/cancers17121997 (PMC12190973; doi:10.3390/cancers17121997)
Supplement: Supplementary file 1 [file cancers-17-01997-s001.zip › cancers-3601110-supplementary.pdf]

Supplementary Table S1. Summary of Study Eligibility for Overall Survival Endpoints and NF1 Stratification

Study				1-y OS	3-y OS	5-y OS	NF1 1-y OS	NF1 3-y OS	NF1 5-y OS	NF1 vs Sporadic HR	Notes / reason for exclusion
**Study**	**1-y**	**3-y**	**5-y**	**1-y**	**3-y**	**5-y**	**NF1 1-y**	**NF1 3-y**	**NF1 5-y**	**NF1 vs Sporadic HR**	**Notes / reason for exclusion**
**Zhu et al., 2011**				✓	✓	✓	✗	✗	✗	✗	OS endpoints reported, but no NF1 subgroup or comparison
**Rastrelli et al., 2017**				✓	✓	✓	✗	✗	✗	✗	OS endpoints reported, but no NF1 subgroup or comparison
**Mowery et al., 2019**				✓	✓	✓	✗	✗	✗	✗	OS endpoints reported, but no NF1 subgroup or comparison
**Sobezuk et al., 2020**				✓	✗	✗	✗	✗	✗	✗	OS endpoints reported, but no NF1 subgroup or comparison
**Roohani et al., 2023**				✓	✓	✓	✗	✗	✗	✗	OS endpoints reported, but no NF1 subgroup or comparison
**Ptchelintseva et al., 2023**				✓	✓	✓	✗	✗	✗	✗	OS endpoints reported, but no NF1 subgroup or comparison
**Rekhi et al., 2008**				✗	✗	✓	✗	✗	✗	✗	OS endpoints reported, but no NF1 subgroup or comparison
**Bishop et al., 2018**				✗	✗	✓	✗	✗	✗	✗	OS endpoints reported, but no NF1 subgroup or comparison
**Stucky et al., 2012**				✗	✗	✓	✓	✓	✓	✓	Included in NF1 vs sporadic HR; stratified outcomes available

**LaFemina et al., 2013**				✗	✗	✗	✓	✓	✓	✓	NF1 & sporadic reported separately; no pooled OS
**Zehou et al., 2013**				✗	✗	✗	✓	✗	✓	✗	NF1-only cohort
**Dunn et al., 2013**				✗	✗	✗	✗	✗	✓	✗	NF1-only cohort
**Martin et al., 2019**				✗	✗	✓	✗	✗	✓	✓	NF1 vs sporadic comparison included; subgroup analysis available

Supplementary Table S2. Demographics, tumor characteristics, presentation, treatment, and outcomes in the included studies

Study	Sample Size	Mean Age	Sex; % Male	% High-grade	Tumor size (cm^3)	Location; Type, %	Sx; Type, %	Genetic Syndrome; Type, %	% Met.	Diagnostic Modality	% Surg.	Conservative; Type, %	% GTR	% Recur.	Follow-up (months)	% Mortality
**Ejerskov et al., 2022**	**19**	Mdn: 31.8	–	–	–	–	–	NF1, 100.0	–	–	57.9	Chemo, 26.3<br>Radiation, 57.9	72.7	–	–	58.0
**Moretti et al., 2011**	**10**	40	60.0	90.0	Mean 10.9	Upper arm, 20.0<br>Hip/Pelvis, 30.0<br>Thigh, 20.0<br>Ankle, 10.0<br>Jaw, 10.0<br>Chest wall, 10.0	–	NF1, 40.0	40.0	–	100.0	Chemo, 100.0<br>Radiation, 70.0	60.0	30.0	Mean: 25.4	–
**Martin et al., 2019**	**714**	–	53.6	–	–	Extremity, 39.1<br>Trunk, 39.5<br>RP, 5.6<br>Head and Neck, 12.5	–	NF1, 25.8	11.8	–	–	–	–	–	–	–
**Zhu et al., 2011**	**14**	44	50.0	71.4	–	C-spine, 28.6<br>T-spine, 35.7<br>L-spine, 28.6<br>Sacral, 7.1	–	NF1, 7.1	42.9	CT, MRI	100.0	Radiation, 7.1	85.7	85.7	Mean: 23.9	71.4

**Roohani et al., 2023**	**42**	Mdn: 48.0	42.1	78.7	≤ 5 cm, 47.6%<br>> 5 cm, 31.0%	Trunk, 15.8<br>Extremity, 22.8<br>Head and Neck, 26.3<br>Thoracic, 10.5<br>RP, 5.3<br>Abdomi−l, 8.8<br>Spi−l, 10.5	–	NF1, 26.3	15.8	CT, MRI	73.7	Chemo, 42.1<br>Radiation, 47.4	78.8	–	Mdn: 20.0	–
**Dunn et al., 2013**	**23**	38.2	65.2	–	Mean 11.1	Trunk/pelvis, 52.2<br>Other, 47.8	Mass, 69.6<br>Pain, 30.4	NF1, 100.0	17.4	CT, MRI, PET, Biopsy	100.0	Chemo, 43.5<br>Radiation, 91.3	65.2	60.9	–	39.0
**Ptchenlintseva et al., 2023**	**13**	43.6	46.2	84.6	Mean 7.9	Lower Extremities, 46.2<br>Trunk, 46.2<br>Head, 15.4	–	NF1, 61.5	7.7	Biopsy, MRI, CT	100.0	Chemo, 15.4<br>Radiation, 84.6	92.3	84.6	Mean: 36.8	50.0
**Ma et al., 2014**	**43**	Mdn: 41	58.1	–	Mean 7.1	Maxilla, 27.9<br>Mandible, 11.6<br>Neck, 11.6<br>Parotid, 9.3<br>Other, 39.5	–	NF1, 30.2	37.2	MRI, CT	100.0	Chemo, 25.6<br>Radiation, 76.7	76.7	51.2	–	53.5
**Mowery et al., 2019**	**2858**	47.0	54.4	68.6	Mdn: 7.5	Trunk and Extremity, 74.3<br>Head and Neck, 12.3 Central Nervous System, Cranial Nerves, and Meninges, 4.4 Overlapping or NOS, 9.0	–	–	13.1	–	66.7	Chemo and/or Radiation, 51.1	74.5	–	Mdn: 30.5	50.0
**Rastrelli et al., 2017**	**20**	41.3	60.0	–	–	Upper limb, 20.0<br>lower limb, 80.0	–	–	–	MRI, CT	100.0	Hyperthermic isolated limb perfusion, 100.0<br>Radiation, 50.0	–	5.0	Mean: 7.2	–
**Rekhi et al., 2008**	**10**	30.0	60.0	80.0	–	Lower limb, 40.0<br>Head and Neck, 20.0 Upper Limb, 10.0<br>Chest wall, 10.0<br>Back, 10.0 RP, 10.0	Enlarging mass, 80.0	NF1, 60.0	30.0	Biopsy	100.0	Chemo, 50.0<br>Radiation, 40.0	–	50.0	–	–

**Zehou et al., 2013**	**21**	Mdn: 31	57.1	95.2	Mean 13.0	Head and Neck, 14.3 Trunk, 19.0 Extremity, 38.1 Abdomen or pelvis, 28.6	Pain, 95.2 Growing mass, 90.5	NF1, 100.0	14.3	CT, MRI	76.2	Chemo, 100.0 Radiation, 40.0	37.5	52.4	–	90.0
**Sobczuk et al., 2020**	**115**	Mdn: 43.1	51.3	69.6	Mdn 12.0	Arm, 13.9 Lower Distal, 13.9 Trunk, 18.3 Head and Neck, 6.1 Visceral, 5.2 Lower Proximal, 31.3 RP, 11.3	–	NF1, 20.0	86.1	CT, MRI	17.4	Chemo, 100.0	84.3	50.4	Mdn: 11.8	–
**Bishop et al., 2018**	**71**	Mdn: 39.0	45.1	67.6	<5 cm, 26.8% > 5 cm, 67.6%	Head and neck, 14.1 Trunk, 21.1 Upper extremities, 19.7 Lower extremities, 45.1	–	NF1, 36.6	90.0	–	100.0	Chemo, 49.0 Radiation, 100.0	56.0	66.2	Mdn: 118.0	61.0
**LaFemi et al., 2013**	**105**	Mdn: 38.0	67.6	100.0	<5 cm, 34.3% 5–10 cm, 28.6% >10 cm, 37.1%	Extremity, 40.0 Abdomen/RP, 28.6 Trunk, 24.8 Head and neck, 6.7	–	NF1, 40.0	–	–	99.0	–	–	61.9	Mdn: 31.2	42.0
**Stucky et al., 2012**	**175**	Mdn: 44.0	48.6	63.4	Mdn 6.0	Head and neck, 19.4 Trunk, 34.3 Extremity, 44.6 Unknown, 1.7	–	NF1, 32.6	19.4	Biopsy	94.9	Chemo, 26.9 Radiation, 60.6	69.1	–	Mdn: 74.0	51.4
