# Supplementary figures and images for: An Assessment of Surgical Outcomes in Malignant Peripheral Nerve Sheath Tumors: A Systematic Review and Meta-Analysis of Surgical Interventions
Source: Cancers (Basel). 2025 Jun 15;17(12):1997. doi: 10.3390/cancers17121997 (PMC12190973; doi:10.3390/cancers17121997)

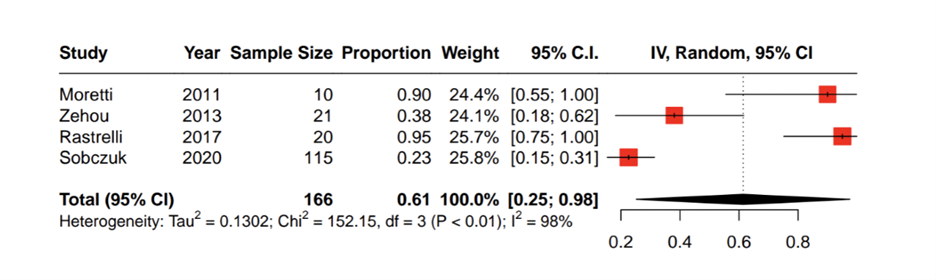

Supplement: Supplementary file 1 [file cancers-17-01997-s001.zip › FigureS1.png]

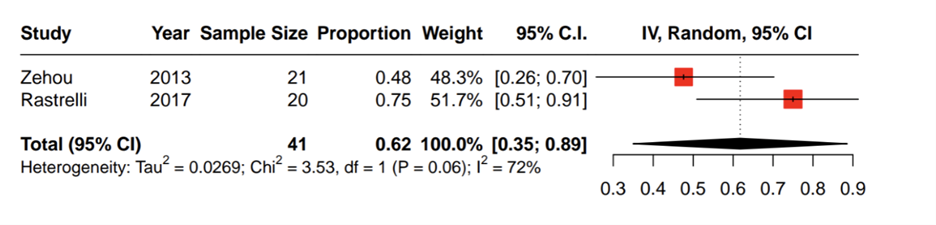

Supplement: Supplementary file 1 [file cancers-17-01997-s001.zip › FigureS2.png]

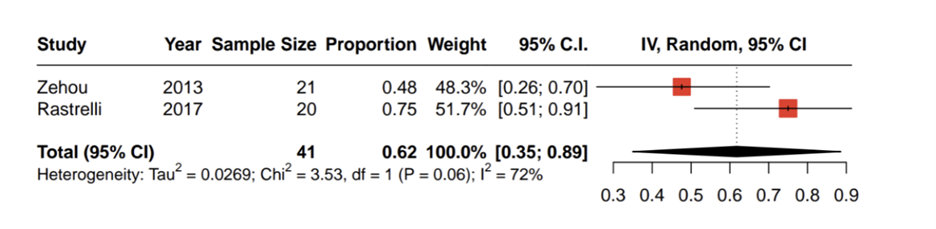

Supplement: Supplementary file 1 [file cancers-17-01997-s001.zip › FigureS3.png]

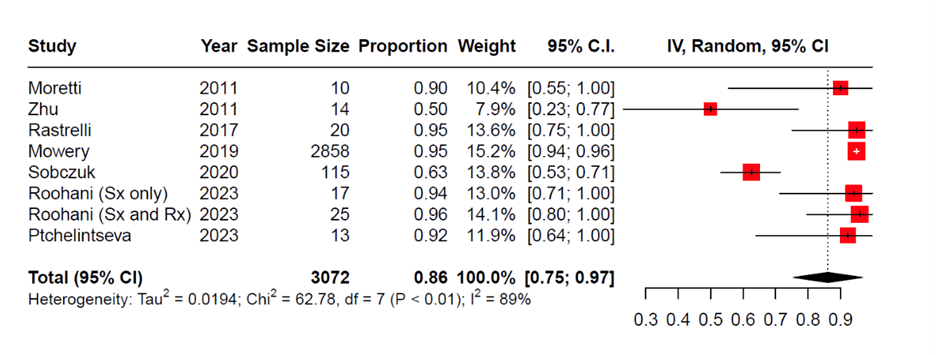

Supplement: Supplementary file 1 [file cancers-17-01997-s001.zip › FigureS4.png]

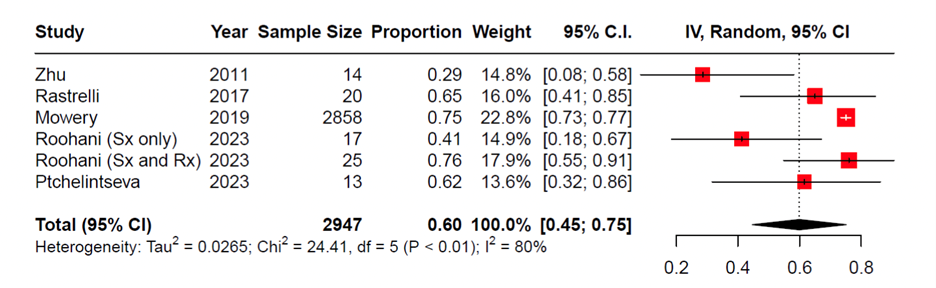

Supplement: Supplementary file 1 [file cancers-17-01997-s001.zip › FigureS5.png]

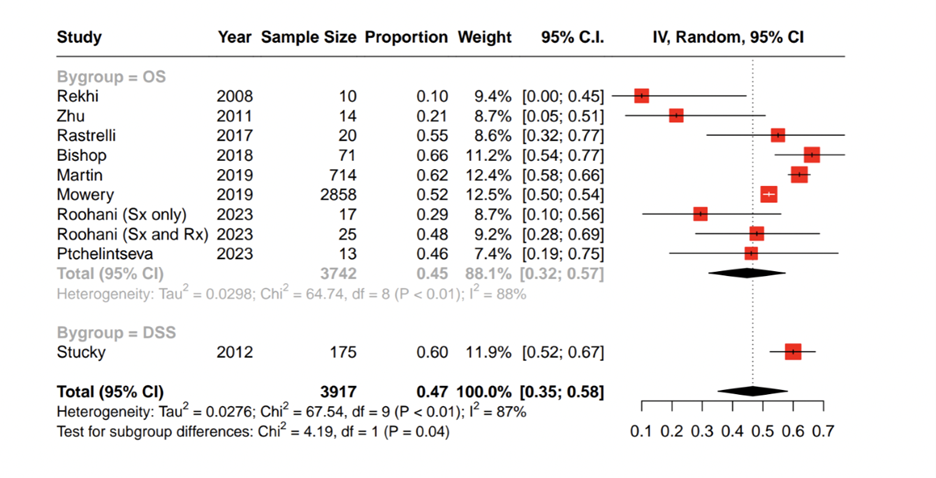

Supplement: Supplementary file 1 [file cancers-17-01997-s001.zip › FigureS6.png]

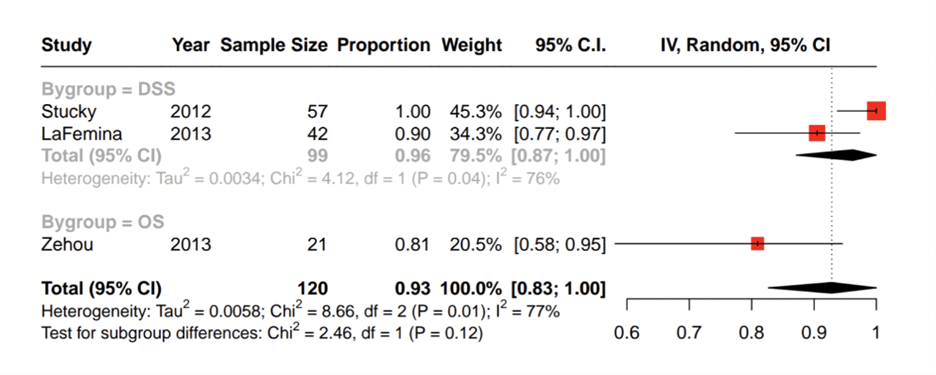

Supplement: Supplementary file 1 [file cancers-17-01997-s001.zip › FigureS7.png]

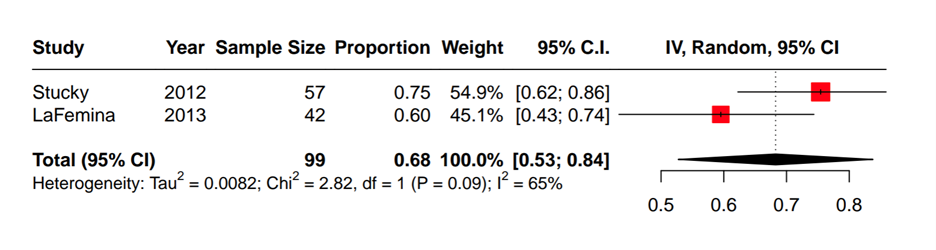

Supplement: Supplementary file 1 [file cancers-17-01997-s001.zip › FigureS8.png]

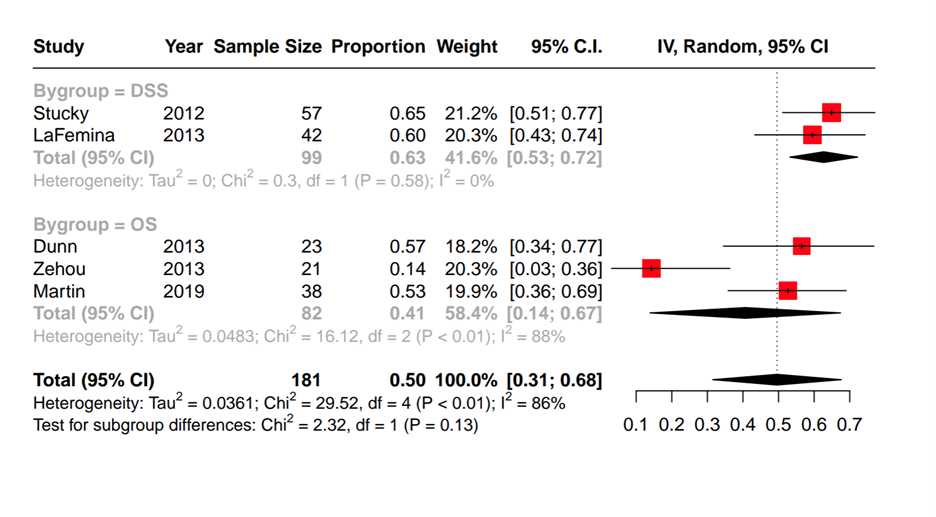

Supplement: Supplementary file 1 [file cancers-17-01997-s001.zip › FigureS9.png]
